# Lightweight Asymmetric Convolutional Residual Network for Efficient Motion Image Deblurring

**DOI:** 10.3390/e28080896

**Published:** 2026-08-10

**Authors:** Hongyin Li, Yang Yue, Jiahao Li, Zhongning Guo, Ming Wu

**Affiliations:** 1 School of AI Engineering, Guangzhou College of Technology and Business, Guangzhou 510006, China; lihongyin@gzgs.edu.cn; 2 School of Electronics and Communication Engineering, Guangzhou University, Guangzhou 510006, China; jiahaoli@e.gzhu.edu.cn; 3 College of Mechanical and Electrical Engineering, Guangdong University of Technology, Guangzhou 510006, China; znguo@gdut.edu.cn; 4 Department of Computer Science, KU Leuven, 3000 Leuven, Belgium; 5 Department of Mechanical Engineering, KU Leuven, 3000 Leuven, Belgium

**Keywords:** lightweight architecture, asymmetric convolution, motion image deblurring

## Abstract

Motion image deblurring remains challenging because many existing models rely on complex architectures, leading to high computational cost and parameter redundancy, particularly under non-uniform blur in real-world scenes. To mitigate these limitations, we propose a Lightweight Asymmetric Convolutional Residual Network (LACR) for efficient motion image deblurring. LACR introduces an asymmetric convolutional residual module that combines local spatial embedding with horizontal and vertical asymmetric refinement, enabling direction-sensitive blur modeling with reduced spatial redundancy. A shallow deep feature fusion mechanism is further designed to integrate low-level convolutional cues with deep restoration representations, thereby complementing low-frequency structural information with high-frequency texture details. Experiments on four benchmark datasets show that LACR improves the reconstruction of edges, textures, and structural details while maintaining a lightweight design. Compared with representative lightweight deblurring methods under consistent evaluation settings, LACR achieves an average PSNR gain of 0.38 dB and reduces computational cost by more than 20%. Quantitative and qualitative results demonstrate that LACR achieves a favorable balance between restoration quality and computational efficiency.

## 1. Introduction

Image deblurring is a fundamental task in computer vision that aims to recover latent sharp images from blurred observations through reconstruction algorithms [1]. The task becomes particularly challenging in practical dynamic scenes, where low illumination, camera shake, and fast object motion can introduce severe blur artifacts [2]. Such degradation not only reduces image quality, but also impairs the extraction of reliable visual information [3], thereby hindering downstream tasks such as object detection and image segmentation [4]. Related studies on spatial distortion and stability detection further indicate that dynamic degradation processes require robust spatial feature modeling to capture structural variations and suppress disturbance-induced artifacts. The above findings motivate the development of efficient deblurring models that preserve structural information while maintaining practical computational efficiency [5].

To address the challenges of dynamic motion blur, deep learning-based methods have been widely investigated for motion blur removal in complex scenes. End-to-end convolutional neural networks (CNNs) have become a mainstream solution because of their strong feature representation and reconstruction capabilities. DeepDeblur [6] is a representative early work that formulated deblurring as an end-to-end restoration problem, promoting the transition from conventional optimization-based approaches to data-driven learning methods [7]. Progressive restoration strategies [8] further improved deblurring performance through multi-stage refinement, providing an important basis for later end-to-end deblurring studies.

With the continuous maturation of the deep learning paradigm, research on image blur shows a rapid development trend. More recently, a large number of new algorithms have emerged in image deblurring, achieving remarkable breakthroughs in visual quality and structural modeling. For instance, in 2018, Kupyn et al. [9] introduced the generative adversarial network (GAN) for the task of image deblurring and proposed DeblurGAN. This approach demonstrated exceptional performance in detail preservation and yielded impressive reconstruction outcomes. Subsequently, in 2019, Kupyn et al. [10] proposed DeblurGAN-v2 on this basis. By improving the generator structure and designing the loss function, better performance was achieved. Therefore, the GAN network design has been proven to effectively improve the recovery performance in the image deblurring task. However, its multi-scale processing mechanism inevitably leads to a significant increase in computational complexity and the number of model parameters, making it impossible for the algorithm to balance computational efficiency.

Given the parameter redundancy and high computational complexity of image deblurring models, several optimization strategies have been explored [11,12]. Lightweight architectures [13] and multi-task learning frameworks [14] reduce computational cost by compressing model structures, sharing feature representations, or introducing auxiliary learning objectives, while improving reconstruction quality to some extent. In parallel, high-capacity models such as Stripformer [15] and SFNet [16] further improve restoration fidelity through long-range dependency modeling and selective frequency representation, but their complex feature interactions may increase deployment cost and limit practical efficiency. Therefore, efficient designs that better balance restoration quality, parameter scale, and computational complexity are still needed.

Inspired by U-Net, Guo et al. [17] designed LNNet to improve inference efficiency by reducing parameter size and computational complexity. Cho et al. [18] further proposed MIMO-UNet, which employs a multi-input and multi-output structure to enhance multi-scale restoration [19]. These methods demonstrate the effectiveness of lightweight design and multi-scale modeling for image deblurring. However, existing efficient deblurring networks still face two limitations. First, compact architectures may weaken the modeling of direction-sensitive motion blur and fine texture structures. Second, multi-scale features are often fused in a generic manner, which may be insufficient for complementing shallow structural cues with deeper restoration representations.

**Figure 1:**
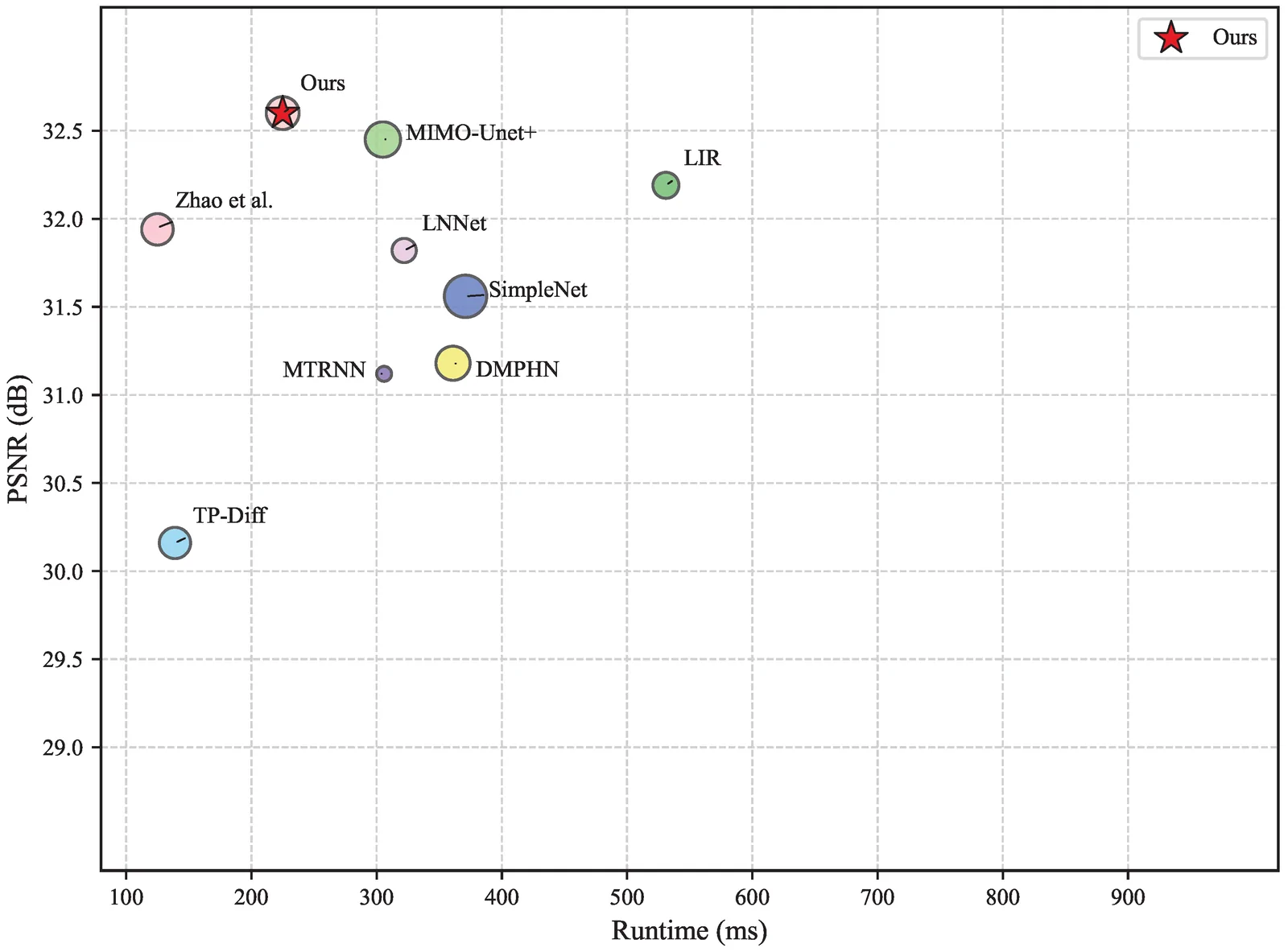
Comparative analysis of representative motion deblurring methods on the GoPro dataset. The y-axis denotes PSNR (dB), the x-axis denotes runtime (ms), and the circle size represents the number of model parameters.

Motivated by the above limitations, we propose a Lightweight Asymmetric Convolutional Residual Network (LACR) with multiple inputs and multiple outputs for efficient motion image deblurring. Different from directly stacking lightweight convolutional blocks, LACR introduces an Asymmetric Convolutional Residual Module (ACRM) to combine local spatial embedding with horizontal and vertical asymmetric refinement for direction-sensitive blur modeling. In addition, an Asymmetric Feature Fusion Module (AFFM) is designed to integrate shallow convolutional features with ACRM-extracted representations, allowing low-frequency structural information and high-frequency texture details to be jointly enhanced. As shown in Figure 1, LACR achieves competitive PSNR with moderate computational complexity on the GoPro dataset, indicating a favorable trade-off between restoration quality and efficiency. The network has three key characteristics:

(i) We propose LACR, a parameter-efficient multi-branch network for motion image deblurring. Different from directly stacking lightweight convolutional blocks, LACR organizes asymmetric residual refinement and cross-branch feature aggregation within a multi-scale restoration framework, enabling effective recovery of direction-sensitive blur structures under moderate model complexity.
(ii) We design an Asymmetric Convolutional Residual Module (ACRM) as the main refinement component. ACRM retains local spatial embedding through a 3×3 convolution and introduces horizontal and vertical asymmetric refinement within a residual structure, reducing redundant spatial computation while preserving structural boundaries and fine texture details.
(iii) Extensive experiments on four public benchmarks demonstrate the effectiveness of LACR on synthetic and real-world motion blur. Quantitative comparisons, complexity analysis, visual results, and ablation studies show that LACR achieves competitive restoration quality with a favorable trade-off between model complexity and computational efficiency.

## 2. Related Work

This section presents recent advances in motion image deblurring and lightweight networks.

### 2.1. Motion Image Deblurring

In motion image deblurring, improving reconstruction quality while controlling computational cost remains a central challenge. Early deep deblurring methods employed multi-scale convolutional restoration strategies and pixel-level aligned datasets to recover latent sharp images [6,20]. GAN-based approaches further introduced adversarial learning to model blur degradation and sharp image reconstruction jointly [9,10]. Recurrent and progressive architectures, such as MTRNN [21], improved restoration by capturing dependencies across multiple stages or scales. These methods have advanced end-to-end deblurring, but they often rely on deeper networks [22], recurrent inference, or multi-stage processing. The resulting parameter redundancy and computational burden limit practical deployment under constrained memory and computation budgets.

To improve efficiency, lightweight multi-scale deblurring networks have been developed. For example, MIMO-UNet+ [18] strengthens spatial representation through multi-scale input and output branches. Meanwhile, LNNet [17] reduces model complexity using a compact nested architecture. Such methods demonstrate that compact design reduces computational cost while maintaining restoration quality. However, many lightweight networks still rely on generic convolutional simplification or basic feature fusion. They give limited attention to the directional characteristics of motion blur and the feature interactions between shallow structural cues and deeper restoration layers. As a result, fine textures and local edge structures may still be degraded after deblurring, particularly in text, contour, and dense-pattern regions. Such restoration challenges call for a task-tailored lightweight architecture that can preserve direction-sensitive blur information while reducing redundant computation.

Recent image restoration studies have introduced attention mechanisms and state space models to enhance global dependency modeling and sequential feature representation. Concertormer [23] uses transformer-style feature interaction for image deblurring, while MaIR [24] and EAMamba [25] explore Mamba-based representations to improve restoration performance. Although such models achieve strong reconstruction capability, global interaction and state-space modeling may increase deployment complexity. In contrast, LACR adopts a convolution-friendly lightweight design that combines asymmetric residual refinement with shallow-deep feature fusion, aiming to recover direction-sensitive blur structures and fine visual details under moderate computational cost.

### 2.2. Lightweight Networks

Model efficiency has become an important consideration in deep image deblurring, especially for resource-constrained visual restoration scenarios [17]. Nevertheless, preserving reconstruction fidelity under a compact parameter and computation budget remains challenging [18,26]. Efficient convolutional designs, such as grouped convolution and asymmetric convolution, provide feasible ways to reduce redundant spatial computation [27,28]. In particular, low-rank kernel decomposition was introduced by Rigamonti et al. [29] and later adopted in efficient real-time networks such as ERFNet [30]. These studies demonstrate the effectiveness of factorized convolution for reducing parameters and computation. However, most existing applications of asymmetric convolution focus on high-level recognition or segmentation tasks, where semantic abstraction is more important than pixel-level texture fidelity. In contrast, motion image deblurring requires accurate recovery of local edges, structural boundaries, and high-frequency details, making the direct transfer of such efficient designs non-trivial [31].

For low-level restoration, directly replacing standard convolutions with factorized or grouped operations may weaken local structure modeling and high-frequency detail recovery. Previous lightweight restoration networks, such as ESDNet [32], have used residual learning, efficient convolution, and feature fusion to reduce model complexity. Although these operations have been widely explored, their task-specific organization for motion image deblurring remains important. The distinction of LACR lies in how these operations are organized and adapted for direction-sensitive blur restoration. Specifically, ACRM retains local spatial embedding and introduces horizontal and vertical asymmetric refinement within a residual restoration path to model motion blur patterns. AFFM further fuses shallow convolutional features with ACRM-extracted representations, allowing structural cues and texture details to complement each other during reconstruction. This task-oriented organization enables LACR to reduce redundant computation while maintaining the visual information required for high-quality deblurring.

Motion-aware deblurring has also been studied from multi-modal and multi-frame perspectives, including event-based restoration, local-motion modeling [33], and dual-lens/video deblurring [34]. These methods introduce useful motion priors and can improve restoration quality in challenging dynamic scenes. However, they often depend on additional sensors, multiple input frames, or increased computational cost, which may limit their applicability in standard single-image restoration settings. In contrast, LACR focuses on lightweight single-image motion deblurring through a convolution-friendly design. By avoiding extra sensing inputs and complex sequential modeling, LACR aims to balance restoration quality, model compactness, and computational efficiency within a practical and easily deployable restoration framework.

## 3. Proposed Method

The proposed LACR network is a U-Net-based multi-scale architecture designed to restore sharp latent images from blurred inputs. The framework is hierarchically organized into Encoder Modules (EMs) and Decoder Modules (DMs), and integrates four main components: the Shallow Convolutional Module (SCM), the Feature Attention Module (FAM), the Asymmetric Feature Fusion Module (AFFM), and the Asymmetric Convolutional Residual Module (ACRM). SCM extracts low-level spatial features from the input images, while FAM recalibrates informative feature responses within the encoder. ACRM serves as the main feature refinement unit in the encoder-decoder structure. In the baseline configuration, the refinement stage adopts 20 stacked Asymmetric Convolutional Residual Blocks (ACRBs), where each ACRB performs asymmetric residual refinement to improve feature representation with moderate computational cost. AFFM acts as a cross-scale fusion bridge between the encoder and decoder, integrating shallow structural cues with deeper restoration features to enhance multi-scale feature interaction.

### 3.1. Network Architecture

**Figure 2:**
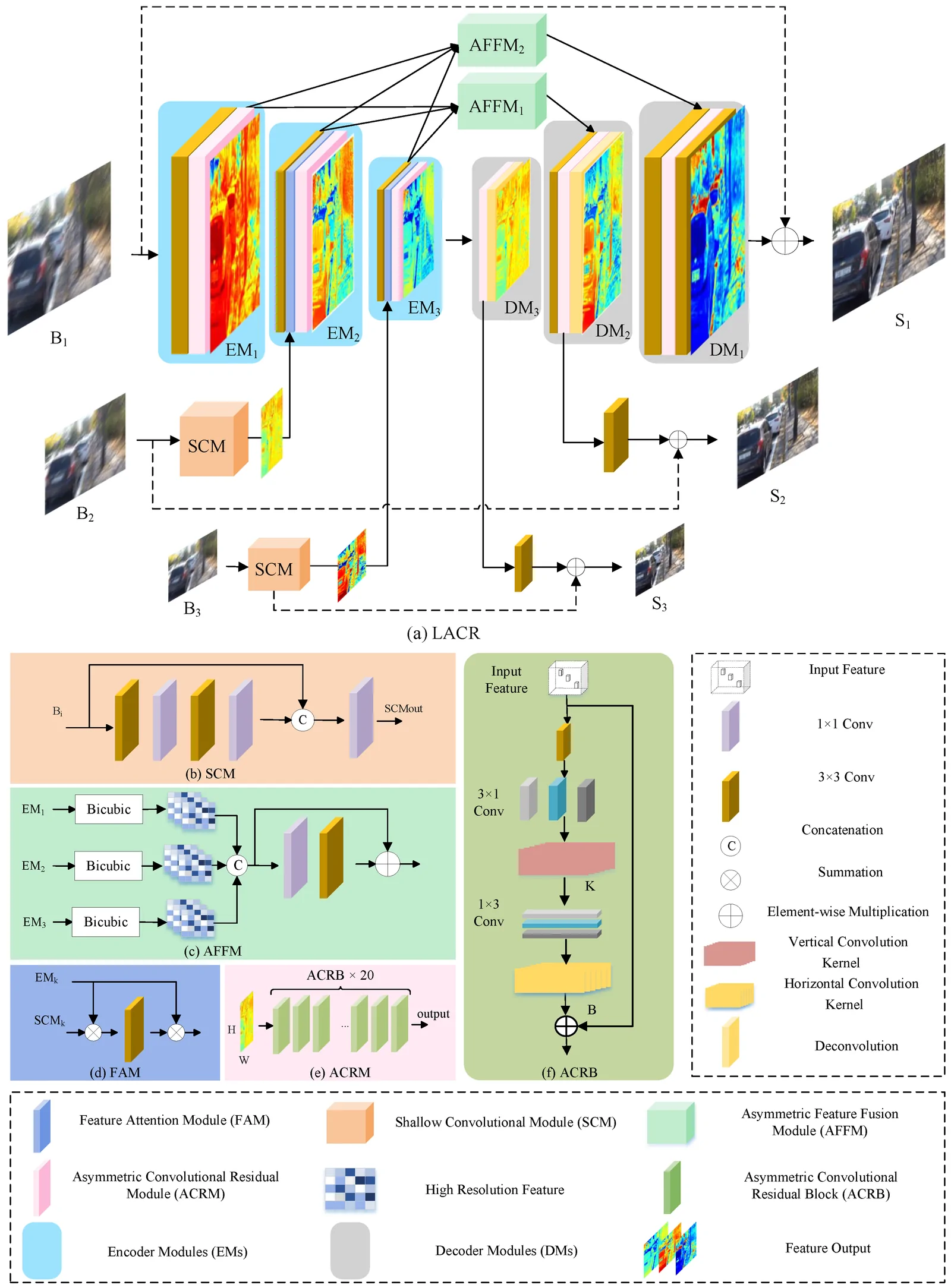
(**a**) Overview of the proposed LACR network. (**b**–**f**) show the main components of LACR: the Asymmetric Feature Fusion Module (AFFM), Shallow Convolutional Module (SCM), Feature Attention Module (FAM), and Asymmetric Convolutional Residual Module (ACRM). The light-blue rounded boxes denote the Encoder Modules (EMs), where each EM contains a 3×3 convolutional embedding layer and stacked ACRM blocks, with stride-2 convolution used for downsampling in EM_1_ and EM_2_. The light-gray rounded boxes denote the Decoder Modules (DMs), where stacked ACRM blocks are used for feature refinement. DM_3_ and DM_2_ use deconvolution for upsampling, while DM_1_ uses two 3×3 convolution layers for final feature reconstruction.

As shown in Figure 2a, LACR adopts a multi-scale input strategy, where the blurred image and its downsampled versions with scales of 1/2 and 1/4 are fed into different network levels. Besides the hierarchical features generated during encoding, SCM extracts shallow spatial and channel features from the downsampled inputs $B_{2}$ and $B_{3}$. These shallow features are then fused with encoder features through FAM, enabling the network to preserve low-level structural cues that may be weakened by repeated downsampling. Compared with a conventional U-Net that mainly relies on encoder-derived features, this multi-scale input and feature fusion strategy provides more complementary information for motion deblurring while maintaining computational efficiency.

The structure of SCM is shown in Figure 2b. To maintain computational efficiency, SCM consists of two 3×3 convolution layers and two 1×1 convolution layers. The 3×3 convolutions extract shallow spatial features, while the 1×1 convolutions adjust channel dimensions and reduce redundant feature responses. Let $SCM_{k}^{out}$ denote the output of SCM at the *k*-th scale. For the second and third network levels, $SCM_{k}^{out}$ is fused with the corresponding encoder feature through FAM. Specifically, FAM performs element-wise feature recalibration and then applies a 3×3 convolution to refine the fused representation. The effectiveness of this fusion strategy is verified in Section 4.3, where SCM and FAM improve restoration performance compared with generic feature fusion.

As shown in Figure 2d, FAM is introduced to recalibrate multi-scale features and enhance informative responses, thereby improving restoration accuracy. SCM extracts shallow spatial and channel features from the downsampled inputs, providing complementary low-level information for feature fusion. The effectiveness of SCM and FAM is further verified in the ablation study described in Section 4.5. Within each EM, a 3×3 convolution is first applied for local feature embedding and channel projection, followed by ACRM based asymmetric residual refinement. For EM_1_ and EM_2_, stride-2 convolution is used for downsampling to reduce spatial resolution and enlarge the receptive field.

In the LACR network, Decoder Modules (DMs) at different scales are used to generate multi-scale restored outputs. Each scale is supervised by its corresponding sharp image, which encourages the network to progressively refine blurred structures from coarse to fine. The image reconstruction at the *k*-th scale is formulated as:

(1) $${\hat{S}}_{k}=o\;\left(DM_{k}\left(X_{k}\right)\right)+B_{k},\;k=1,\ldots ,3$$

where ${\hat{S}}_{k}$ denotes the restored image at the *k*-th scale, $B_{k}$ is the blurry input image at the same scale, and o(·) represents the output projection layer. $X_{k}$ denotes the input feature of $DM_{k}$. For k=1,2, $X_{k}$ is obtained by combining the corresponding AFFM output with the feature propagated from the next decoder module. For k=3, $X_{k}$ is obtained from the output of the encoder module.

(2) $$X_{k}=\left\{\begin{array}{cc} (AFFM_{k}^{out},\;DM_{k+1}^{out}), & k\in \{1,2\},\\ EM_{k}^{out}, & k=3. \end{array}\right.$$

In Equation (2), ${\mathrm{AFFM}}_{k}^{out}$, ${\mathrm{EM}}_{k}^{out}$, and ${\mathrm{DM}}_{k}^{out}$ denote the outputs of the *k*-th AFFM, EM, and DM, respectively. The operator (·,·) represents feature aggregation with convolutional fusion, providing the input feature $X_{k}$ for the corresponding decoder module.

### 3.2. Asymmetric Feature Fusion Module

Traditional coarse-to-fine image deblurring algorithms typically propagate only coarse-scale features to finer-scale networks, limiting the flexibility of information flow across different frequency components. To address this limitation, we introduce an Asymmetric Feature Fusion Module (AFFM), which enables bi-directional feature interaction by leveraging dense connections among intra-scale representations. The proposed AFFM follows recent architectural designs in which feature enhancement and spatial alignment before fusion are used to attenuate noise and blur, thereby reducing signal degradation during cross-scale aggregation.

As shown in Figure 2c, AFFM fuses encoder features from different scales. In LACR, two AFFM instances, denoted as ${\mathrm{AFFM}}_{1}$ and ${\mathrm{AFFM}}_{2}$, share the same structure but operate at different spatial resolutions. For each AFFM, encoder features from multiple levels are resized to the target resolution through upsampling or downsampling. The aligned features are then concatenated along the channel dimension and refined by convolutional layers to generate scale-aware fused representations. The process is formulated as:

(3) $$\begin{array}{cc} \; & AFFM_{1}=\mathrm{Conv}\left(\mathrm{Concat}(EM_{1},EM_{2}↑,EM_{3}↑)\right)\\ \; & AFFM_{2}=\mathrm{Conv}\left(\mathrm{Concat}(EM_{1}↓,EM_{2},EM_{3}↑)\right) \end{array}$$

where up-sampling (↑) and down-sampling (↓) denote upsampling and downsampling operations, respectively. The fused outputs are subsequently delivered to the corresponding decoder modules, enabling each decoder stage to exploit complementary multi-scale information for image restoration.

### 3.3. Asymmetric Convolutional Residual Module

The Asymmetric Convolutional Residual Module (ACRM), shown in Figure 2e, is the main feature refinement component of LACR and consists of stacked Asymmetric Convolutional Residual Blocks (ACRBs), as detailed in Figure 2f. In our implementation, the ACRM used in the main refinement stage contains 20 stacked ACRBs. Inspired by low-rank kernel decomposition [29], each ACRB retains the first 3×3 convolution of a conventional residual block for local spatial embedding and channel projection, while factorizing the second 3×3 convolution into sequential 1×3 and 3×1 asymmetric convolutions for efficient spatial refinement. The resulting hybrid design reduces redundant computation while preserving local texture and structural information. The asymmetric decomposition is formulated as:   

(4) $$s_{i}*x\approx v_{i}*\left(h_{i}*x\right)$$

where *x* denotes the input feature map, $s_{i}\in R^{d\times d}$ is the original 2D convolution kernel, $h_{i}\in R^{1\times d}$ and $v_{i}\in R^{d\times 1}$ denote the horizontal and vertical 1D convolution kernels, respectively, and ∗ represents convolution.

A standard d×d convolution requires a computational complexity of $O(d^{2}HWC^{2})$, whereas its asymmetric counterpart reduces the complexity to $O(2dHWC^{2})$. Nevertheless, applying such decomposition to all convolutional layers may impair local texture modeling, which is essential for pixel-level restoration tasks. Therefore, the proposed Asymmetric Convolutional Residual Block (ACRB) adopts a hybrid design that retains the initial 3×3 convolution and factorizes only the subsequent 3×3 convolution. The feed-forward process of ACRB is formulated as follows:

(5) $$\begin{array}{c} W_{n}*z=\sum_{c=1}^{C}W_{n}^{c}*z^{c} \end{array}$$

where $W_{n}$ denotes n the convolution kernel, $W_{n}^{c}$ corresponds to the kernel associated with the $\;c_{th}$ input channel among C channel, z is the input feature map, and $\;z^{c}$ denotes its $\;c_{th}$ channel. The resulting approximate convolution is formulated as follows:

(6) $$\begin{array}{cc} W_{n}*z & \approx h_{n}*V=\sum_{k=1}^{K}h_{n}^{k}*V^{k}=\sum_{k=1}^{K}h_{n}^{k}*\left(v_{k}*z\right)=\sum_{c=1}^{C}\left[\sum_{k=1}^{K}h_{n}^{k}*v_{k}^{c}\right]*z^{c} \end{array}$$

The convolution is formulated as a linear combination of separable kernels $\;h_{k}^{n}*v_{k}^{c}$. The general complexity consists of vertical and horizontal convolutions, both with complexity $\;O(KCdH^{\prime }W)$. The result in an overall cost of $\;O(K\left(N+C\right)dH^{\prime }W)$, which is substantially lower than the complexity $\;O(KCd^{2}H^{\prime }W)$ of standard convolution. When K(N+C)<<NCd, the proposed formulation offers significant computational savings.

The separable convolutional approximation is obtained by minimizing the reconstruction error between the original convolution kernels and their low-rank decomposed representations, thereby enabling the factorized filters to approximate the response of the full convolutional kernels as accurately as possible. The corresponding optimization objective is formulated as:

(7) $$\min_{\left\{h_{n}\right\},\left\{v_{k}\right\}}\sum_{n=1}^{N}\sum_{c=1}^{c}{∥W_{n}-\sum_{k=1}^{K-1}h_{n}^{k}*v_{k}^{c}∥}_{2}^{2}$$

From Equation (7), it can be inferred that modeling the separability of convolution with two variables helps eliminate the sum norm constraint. Additionally, by alternately conducting conjugate descent optimization between the horizontal and vertical filter groups, the optimal solution can be achieved. Data reconstruction optimization aims to learn separable bases by minimizing the reconstruction error of the original convolution kernels. However, such an approach does not necessarily lead to optimal CNN prediction performance. As an alternative, the separable bases can be optimized by reconstructing the output responses of the original convolutional layer given the training data. The corresponding data reconstruction formulation is defined as follows:

(8) $$\min_{\left\{h_{n}^{k}\right\},\left\{v_{k}^{c}\right\}}\sum_{i=1}^{\left|x\right|}\sum_{n=1}^{N}{∥W_{n}*\Phi_{l-1}\left(x_{i}\right)-\sum_{c=1}^{C}\sum_{k=1}^{K}h_{n}^{k}*v_{k}^{c}*\Phi_{l-1}\left(x_{i}\right)∥}_{2}^{2}$$

where *l* denotes the index of the convolutional layer, $\Phi_{l-1}\left(x_{i}\right)$ represents the output feature map of the (l−1)-th layer for the input sample $x_{i}$, and $x={\left\{x_{i}\right\}}_{i=1}^{\left|x\right|}$ denotes the training dataset with |x| samples. *N* denotes the number of output channels (i.e., convolution kernels) in the layer. The separable bases are first initialized and incorporated into the network to approximate the original convolution. The approximated layer is then optimized by minimizing the loss $l_{2}$ between the outputs of the original and approximated layers through backpropagation.

### 3.4. Loss Function

Similar to other multi-scale deblurring networks, LACR takes a blurred image and its downsampled versions as inputs. At each scale, the network reconstructs a sharp image corresponding to the given resolution. Compared with the mean squared error (MSE) loss, the $l_{1}$ loss usually produces sharper visual results and more stable convergence. To constrain restored outputs across different scales, a multi-scale content loss based on the $l_{1}$ norm is adopted. The content loss $L_{\mathrm{cont}}$ is formulated as:

(9) $$L_{\mathrm{cont}}=\sum_{k=1}^{K}\frac{1}{t_{k}}{∥{\hat{S}}_{k}-S_{k}∥}_{1}$$

where *K* denotes the number of output scales, with K=3 in our implementation. ${\hat{S}}_{k}$ and $S_{k}$ represent the restored output and the corresponding ground-truth sharp image at the *k*-th scale, respectively. $t_{k}$ is the number of pixels at the *k*-th scale.

In image enhancement and restoration tasks, spatial and frequency constraints are widely used to improve structural consistency and detail recovery. Since motion blur often suppresses high-frequency components, we introduce the Multi-Scale Frequency Reconstruction (MSFR) loss to reduce frequency-domain discrepancies between restored and ground-truth images. Specifically, MSFR computes the $l_{1}$ distance between the frequency representations of multi-scale restored outputs and their corresponding ground-truth images. The MSFR loss is defined as:

(10) $$\begin{array}{c} L_{\mathrm{MSFR}}=\sum_{k=1}^{K}\frac{1}{t_{k}}{∥F\left({\hat{S}}_{k}\right)-F\left(S_{k}\right)∥}_{1} \end{array}$$

where F(·) denotes the Fast Fourier Transform (FFT), which maps an image from the spatial domain to the frequency domain. *K* denotes the number of output scales, with K=3 in our implementation. The final training objective is defined as:

(11) $$L_{\mathrm{total}}=L_{\mathrm{cont}}+\lambda L_{\mathrm{MSFR}}$$

where λ is a weighting hyperparameter that controls the trade-off between the content loss and the multi-scale frequency reconstruction loss. In our experiments, λ is set to 0.1. For the proposed LACR network, Equation (11) defines the empirical objective optimized over the finite training set. From a statistical learning perspective, the objective can be interpreted as empirical risk and is referred to as the loss function in image restoration.

The overall training procedure is summarized in Algorithm 1, and the principal steps are outlined below:
**Algorithm 1:** Training Procedure of LACR
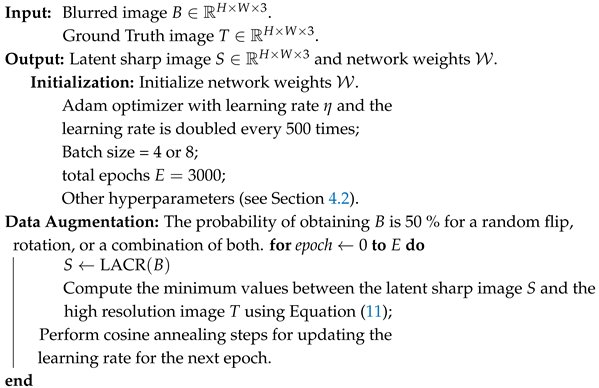


## 4. Experiments

### 4.1. Dataset Details

Experiments were carried out on four common reference datasets, following the methods of [6,35,36]. These datasets are: GoPro [6], a standard image deblurring benchmark, and HIDE [37], featuring blur from the camera-scene motion. Specifically, the RHM-250 dataset [38] (540×960) includes 20,000 training samples, with 2500 images reserved for both validation and testing. For real-world scenarios, the RSCD dataset [39] comprises 4000 images (640×480), which are partitioned into 2500 for training, 750 for validation, and 750 for testing.

### 4.2. Implementation Details

We trained the network on the GoPro dataset for 3000 epochs, sufficient for convergence. A step-wise learning rate schedule is adopted, where the learning rate is decayed by a factor of 0.5 every 500 epochs to facilitate stable optimization. To enhance data diversity and improve model generalization, we adopted random cropping with a patch size of 256 × 256 to introduce spatial variations during training. The preprocessed data was then input into the LACR network, which uses its feature extraction and mapping capabilities for deblurring. The number of workers was set to 4 to accelerate data loading; the learning rate steps were attenuated every 500 epochs, and the gamma was set to 0.5 to adjust the attenuation amplitude, with the objective of achieving efficient model training and good convergence. A fixed random seed was consistently applied across all training and evaluation trials. The number of parameters and computation for each method were calculated based on the input size of (3,224,224). Specifically, all experiments were conducted on an NVIDIA GeForce RTX 4090 GPU (24 GB) running Ubuntu 18.04 using PyTorch 1.7.0.

### 4.3. Performance Comparison

Main Results: We compare LACR with representative image deblurring methods in terms of restoration accuracy and computational efficiency. As shown in Table 1, LACR achieves competitive quantitative performance while maintaining moderate model complexity. Compared with DeepDeblur [6], LACR obtains a clear PSNR improvement over its 28.70 dB result with fewer computational requirements. Although LIR [40] achieves slightly higher accuracy in some settings, LACR maintains a favorable balance between restoration performance and computational efficiency.

Compared with lightweight or efficient networks, including MTRNN [21], MIMO-UNet+ [18], SimpleNet [46], LNNet [17], UHDVD [47], Zhao et al. [28], and LIR [40], LACR shows a favorable balance between PSNR/SSIM performance, parameter size, FLOPs, and runtime. In particular, the proposed asymmetric residual refinement and shallow-deep feature fusion help preserve fine textures and structural details under a compact computational budget. Therefore, the performance comparison is discussed not only from the perspective of restoration accuracy, but also from the efficiency requirements of practical lightweight deblurring applications.

Generalization Ability: We further evaluate all methods on the RHM-250 and RSCD datasets to examine the generalization ability of LACR in more realistic scenarios. RHM-250 is collected in real environments using a high-frame-rate camera, making it closer to practical motion blur conditions than synthetic datasets. RSCD is captured with a beam splitter, which helps record camera ego-motion and object motion in dynamic urban scenes. As shown in Table 2, LACR achieves the best restoration performance on both datasets, demonstrating its robustness to diverse real-world blur patterns.

**Figure 3:**
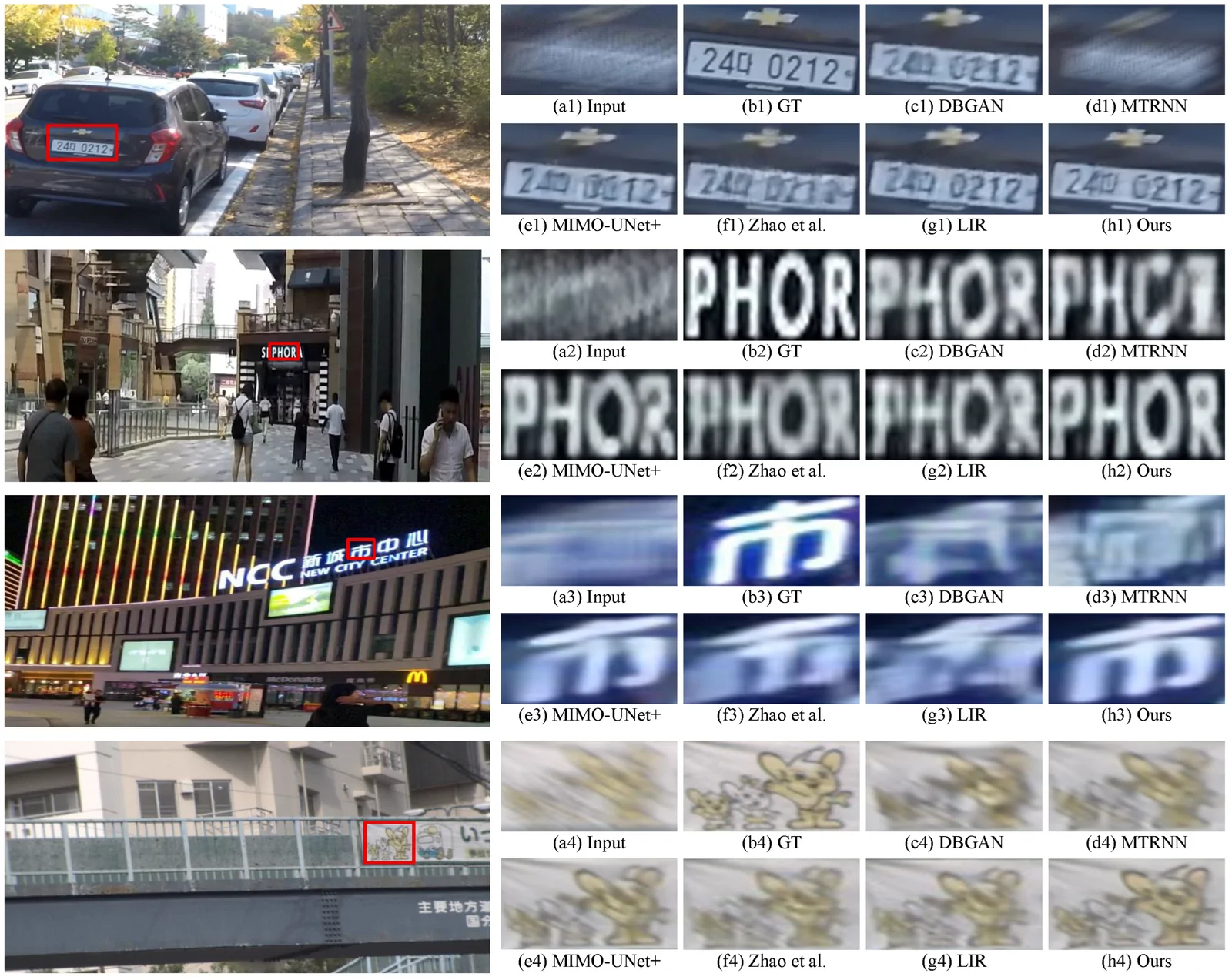
Qualitative comparison of image deblurring performance on the GoPro, HIDE, RHM-250, and RSCD datasets. From top to bottom, the four rows correspond to GoPro, HIDE, RHM-250, and RSCD, respectively. In each row, the leftmost large image shows the full blurry input, and the red box marks the cropped region of interest. Panels (**a1**–**a4**) show the cropped blurry inputs; (**b1**–**b4**), the corresponding ground-truth (GT) sharp images; and (**c1**–**c4**), (**d1**–**d4**), (**e1**–**e4**), (**f1**–**f4**), (**g1**–**g4**), and (**h1**–**h4**), the results produced by DBGAN [44], MTRNN [21], MIMO-UNet+ [18], Zhao et al. [28], LIR [40], and Ours (the proposed LACR framework), respectively. All cropped patches are extracted from the same spatial window for fair comparison.

Qualitative Comparison: Figure 3 presents representative examples from the GoPro, HIDE, RHM-250, and RSCD datasets. The cropped blurry inputs and corresponding ground-truth (GT) sharp images are shown in Figure 3(a1–a4) and Figure 3(b1–b4), respectively. DBGAN [44] in Figure 3(c1–c4) restores part of the structure but leaves noticeable residual blur. MTRNN [21] in Figure 3(d1–d4) improves restoration quality, but some local details remain over-smoothed. MIMO-UNet+ [18] in Figure 3(e1–e4) further enhances sharpness, although artifacts remain around complex edges and text regions. Zhao et al. [28] and LIR [40], shown in Figure 3(f1–f4) and Figure 3(g1–g4), improve detail recovery and noise suppression, but some text contours remain indistinct. In contrast, the proposed LACR in Figure 3(h1–h4) better preserves fine textures, edges, and character contours, yielding clearer and more coherent reconstruction results.

**Figure 4:**
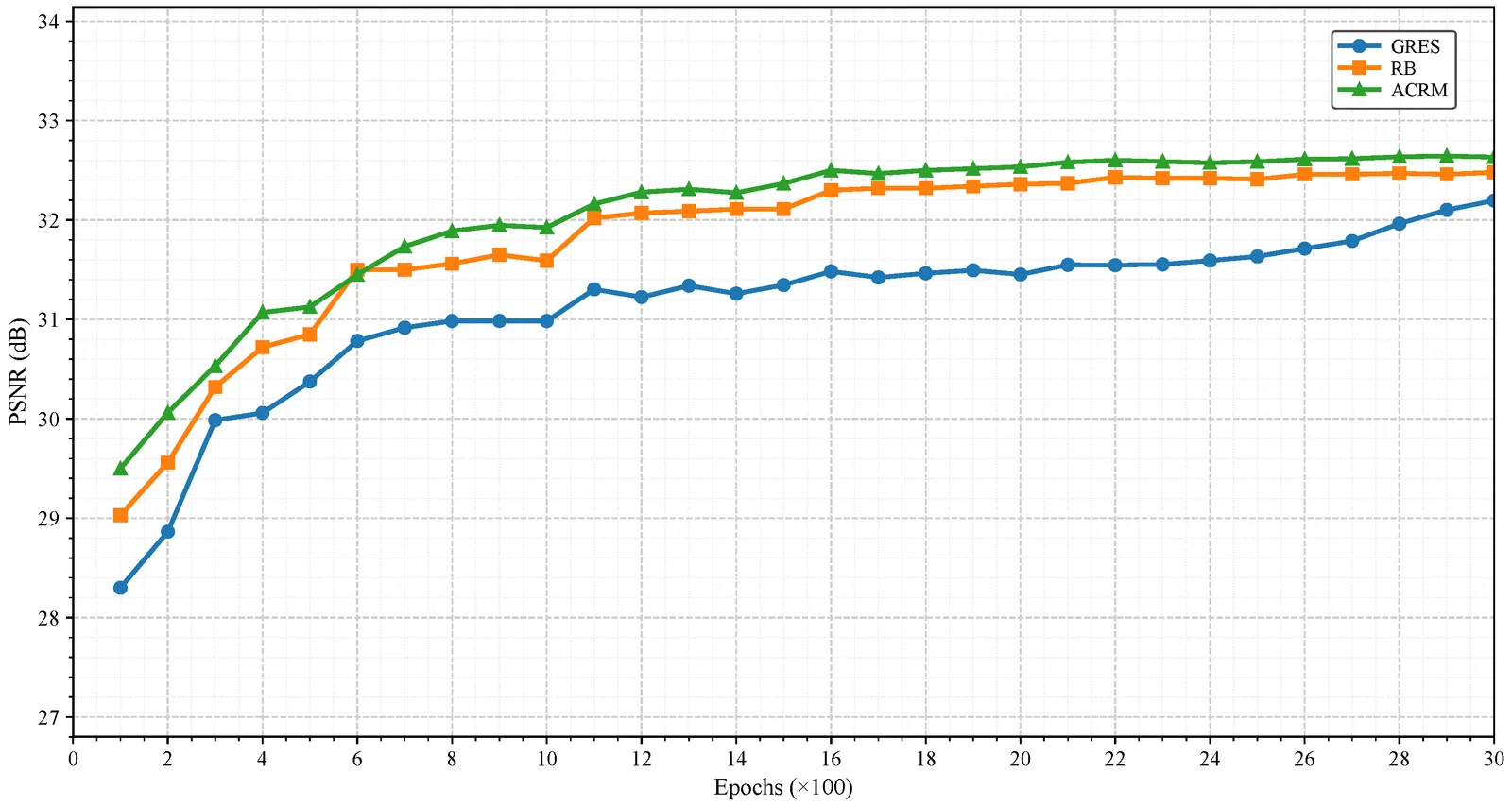
The performance curve serves as a function of epochs at different stages, and the performance of the module is verified using GRES, RB, and ACRM.

Training Time: The convergence analysis evaluates the training behavior of different refinement modules in LACR. We conducted 3000 training iterations on the GoPro dataset and compared ACRM with several baseline module designs, including Residual Block (RB) [18] and Group-Residual (GRES). As shown in Figure 4, LACR exhibits faster convergence and better early-stage training stability, indicating the effectiveness of the proposed asymmetric residual refinement.

### 4.4. Efficiency Analysis

To further evaluate the applicability of the proposed method in real-world scenarios, the RHM-250 and RSCD datasets are used for qualitative analysis. As shown in the third and fourth rows of Figure 3, the cropped blurry inputs and corresponding ground-truth (GT) sharp images are shown in Figure 3(a3,a4) and Figure 3(b3,b4), respectively. In these challenging scenes containing text, character contours, and dynamic motion blur, the proposed LACR, shown in Figure 3(h3,h4), reconstructs structural edges and fine details with improved clarity by integrating AFFM-based feature fusion with ACRM-based residual refinement, while reducing visible artifacts. Compared with representative methods, including DBGAN [44] in Figure 3(c3,c4), MTRNN [21] in Figure 3(d3,d4), MIMO-UNet+ [18] in Figure 3(e3,e4), Zhao et al. [28] in Figure 3(f3,f4), and LIR [40] in Figure 3(g3,g4), LACR shows more favorable visual sharpness and structural preservation.

Table 3 reports the quantitative comparison on the GoPro dataset. The runtime, number of parameters, and FLOPs of all compared methods were measured in our experimental environment using the corresponding available models under the same hardware setting. Except for SimpleNet [46] and DMPHN [36], whose PSNR values were taken from the original papers, the PSNR results of the remaining methods were obtained from our own evaluation. The unified evaluation protocol ensures a consistent comparison of restoration accuracy and computational efficiency.

The efficiency–restoration trade-off of LACR is further quantified in Table 3. Relative to MIMO-Unet+ [18], LACR improves the average PSNR by 0.18 dB while requiring lower FLOPs and a shorter runtime under the same evaluation setting. In comparison with the recent lightweight methods LIR [40] and TP-Diff [48], LACR achieves PSNR gains of 0.44 dB and 2.47 dB, respectively, although LIR reports fewer parameters and lower FLOPs. Compared with DeepDeblur [6], LACR reduces runtime and FLOPs by over 75% and 90%, respectively, while improving PSNR by 3.93 dB. Although TP-Diff [48] attains a lower latency of 139 ms, its restoration accuracy remains inferior to that of LACR. Overall, the quantitative comparison indicates that LACR maintains a more balanced performance profile for lightweight motion deblurring, where restoration fidelity, model compactness, runtime, and computational cost must be jointly considered in practical deployment scenarios.

### 4.5. Ablation Study

To systematically evaluate the contribution of each component in LACR, ablation studies are conducted on the GoPro dataset under the same experimental setting. As shown in Table 4, the baseline model achieves 30.95 dB PSNR. Introducing SCM, AFFM, and RB individually improves PSNR to 31.16 dB, 31.33 dB, and 31.45 dB, respectively, indicating that shallow feature extraction, asymmetric feature fusion, and residual refinement all contribute to image restoration. When SCM and AFFM are combined with different refinement blocks, the configuration with RB reaches 32.45 dB, while replacing RB with GRES obtains 32.15 dB. In contrast, replacing RB with the proposed ACRM achieves the best PSNR of 32.63 dB. Compared with the RB-based complete configuration, ACRM further improves PSNR by 0.18 dB, demonstrating that asymmetric residual refinement is more effective for restoring motion-blurred structures and fine details. The ablation study confirms the complementary role of SCM and AFFM and further verifies the effectiveness of ACRM as the main refinement module in LACR.

The interaction between ACRM and the MSFR loss is further analyzed in Table 5. The baseline model trained with only the content loss achieves 28.20 dB PSNR. Introducing the MSFR loss alone improves PSNR to 28.65 dB, indicating that frequency-domain supervision facilitates the recovery of high-frequency details. Adding ACRM independently increases PSNR more substantially to 32.15 dB, demonstrating its effectiveness in strengthening feature representation for motion deblurring. Combining ACRM with the MSFR loss further raises the performance to 32.63 dB, yielding a gain of 4.43 dB over the baseline. The ablation results indicate that ACRM provides the primary improvement in restoration quality, while the MSFR loss further complements spatial reconstruction by enhancing frequency-domain consistency and fine-detail recovery.

To further isolate the contribution of the supervision terms, Table 6 compares different loss configurations. The model trained only with the content loss achieves 32.31 dB PSNR. Using only the MSFR loss yields a lower PSNR of 32.06 dB, indicating that frequency-domain supervision cannot fully replace spatial-domain reconstruction constraints. Combining the content loss with the MSFR loss increases PSNR to 32.63 dB, corresponding to gains of 0.32 dB and 0.57 dB over the content-only and MSFR-only settings, respectively. These results indicate that the MSFR loss is more effective as a complementary constraint, improving frequency-domain consistency and fine-detail recovery when used together with spatial reconstruction supervision.

The effect of cascade depth in the refinement stage is further examined through an ablation study with four settings, D∈{8,16,20,22}, where *D* denotes the number of stacked ACRBs. As reported in Table 7, increasing *D* from 8 to 20 leads to a consistent and stable improvement in restoration performance. In particular, the PSNR rises from 31.91 dB at D=16 to 32.63 dB at D=20, corresponding to a gain of 0.72 dB. A further increase to D=22 does not yield additional improvement, as the PSNR slightly decreases to 32.62 dB while the model complexity increases to 14.83 M parameters and 123 G FLOPs. These results suggest that excessive cascade depth introduces extra computation with only limited performance benefit. Accordingly, D=20 is adopted as the baseline configuration of LACR, providing a favorable balance between restoration accuracy and computational cost under the current network design.

## 5. Conclusions

Real-time image deblurring requires an effective balance between restoration accuracy and computational efficiency, particularly for mobile devices and resource-constrained applications. The proposed LACR network provides an end-to-end lightweight framework for efficient motion blur restoration. In LACR, AFFM strengthens cross-scale feature interaction, the input feature extraction module provides compact low-level representations, and ACRM performs residual feature refinement through local embedding and asymmetric convolutional operations. The integration of these modules improves texture reconstruction while maintaining moderate model complexity. Experimental results on multiple benchmark datasets demonstrate that LACR achieves competitive deblurring performance with only 13.49 M parameters, indicating a favorable trade-off between restoration quality and efficiency.

Although LACR provides a favorable balance between restoration quality and computational efficiency, several challenging cases remain. Performance may degrade under highly non-uniform or extreme motion blur, large occlusions, fast-moving objects, low-light or noisy inputs, and scenes affected by complex camera shake or rolling-shutter artifacts. In particular, frequency-domain enhancement may amplify noise together with high-frequency details under poor illumination. Future work will investigate adaptive blur-aware modeling and noise-aware frequency regulation to improve robustness under more challenging real-world degradation.

## Figures and Tables

**Table 1:** Comparison results of the representative algorithms on the GoPro and HIDE datasets. Bold values indicate the best performance. The symbol ↑ indicates that higher values are better.

Methods	GoPro (PSNR ↑/SSIM ↑)	HIDE (PSNR ↑/SSIM ↑)
DeepDeblur [CVPR16] [6]	28.70/0.858	24.51/0.872
SRN [CVPR18] [35]	30.26/0.934	28.36/0.915
DMPHN [WACV19] [36]	31.20/0.940	29.09/0.924
MTRNN [CVPR20] [21]	31.15/0.945	29.15/0.918
SVDN [CVPR20] [41]	29.81/0.937	–
SAPH [CVPR20] [42]	31.85/0.948	29.98/0.930
SAPHN+ [CVPR20] [42]	32.02/0.953	–
RADN [AAAI20] [43]	31.76/0.953	29.98/0.930
DBGAN [CVPR20] [44]	31.10/0.942	28.94/0.915
IPT [CVPR21] [45]	32.52/–	–
MIMO-Unet+ [CVPR21] [18]	32.45/0.957	29.90/0.930
SimpleNet [ICCV21] [46]	31.52/0.950	–
UHDVD [ICCV21] [47]	31.33/0.921	23.18/0.752
LNNet [JSA22] [17]	31.70/0.951	29.33/0.922
Zhao et al. [CVPR23] [28]	31.94/0.928	29.67/0.902
LIR [arXiv24] [40]	32.19/0.931	**30.31**/0.917
TP-Diff [ICCV25] [48]	30.16/0.927	27.64/0.892
LACR	**32.63**/**0.961**	30.10/**0.932**

**Table 2:** Comparison of different algorithms on RHM-250 and RSCD datasets. The best performance in each column is highlighted in bold. The symbol ↑ indicates that higher values are better.

Model	RHM-250		RSCD	
	PSNR ↑	SSIM ↑	PSNR ↑	SSIM ↑
MTRNN [21]	26.36	0.816	22.34	0.660
dBGAN [44]	27.89	0.819	20.92	0.611
MIMO-Unet [18]	28.01	0.837	23.85	0.706
MIMO-Unet+ [18]	28.99	0.862	24.49	0.722
UHDVD [47]	26.87	0.810	23.83	0.702
Zhao et al. [28]	28.09	0.833	23.87	0.702
LIR [40]	28.30	0.835	23.38	0.691
TP-Diff [48]	27.46	0.853	23.51	0.721
LACR	**29.45**	**0.870**	**24.84**	**0.731**

**Table 3:** Comparison of parameter count, runtime, and computational complexity of representative deblurring methods. Bold values indicate the best performance in each column.

Methods	PSNR ↑	Runtime (ms) ↓	# Parameters (M) ↓	FLOPs (G) ↓
DeepDeblur [6]	28.70	941	11.7	1023
DMPHN [36]	31.20	361	21.7	309
MIMO-Unet+ [18]	32.45	305	16.11	117
LNNet [17]	31.70	325	**5.94**	55
SimpleNet [46]	31.52	376	35.08	304
Zhao et al. [28]	31.94	**148**	12.18	**35**
LIR [40]	32.19	193	7.31	62.5
TP-Diff [48]	30.16	139	11.89	106
LACR	**32.63**	225	13.49	97

↑ and ↓ indicate that higher and lower values are better, respectively. # denotes the number of parameters.

**Table 4:** Ablation study on different module combinations. Bold values indicate the best performance.

Configuration	SCM	AFFM	RB/GRES/ACRM	PSNR ↑
Baseline				30.95
Baseline + SCM	✔			31.16
Baseline + AFFM		✔		31.33
Baseline + RB			✔ (RB)	31.45
Baseline + SCM + AFFM + RB	✔	✔	✔ (RB)	32.45
Baseline + SCM + AFFM + GRES	✔	✔	✔ (GRES)	32.15
Baseline + SCM + AFFM + ACRM	✔	✔	✔ (ACRM)	**32.63**

↑ indicates that higher values are better. ✔ indicates that the corresponding module is included.

**Table 5:** Interaction between ACRM and MSFR loss. Bold values indicate the best performance.

Configuration	ACRM	MSFR Loss	PSNR ↑
Baseline (content)			28.20
Baseline + MSFR Loss		✔	28.65
Baseline + ACRM	✔		32.15
Baseline + ACRM + MSFR Loss	✔	✔	**32.63**

↑ indicates that higher values are better. ✔ indicates that the corresponding component is included.

**Table 6:** Ablation study on different loss functions. Bold values indicate the best performance.

Loss Setting	Content Loss	MSFR Loss	PSNR ↑
Content only	✔		32.31
MSFR only		✔	32.06
Content + MSFR	✔	✔	**32.63**

↑ indicates that higher values are better. ✔ indicates that the corresponding component is included.

**Table 7:** Ablation study on the cascade depth *D* of ACRM units within the refinement stage. Bold values indicate the best performance.

Configurations	PSNR (↑)	# Parameters (M) (↓)	FLOPs (G) (↓)
LACR (D=8)	31.60	**5.78**	**41.40**
LACR (D=16)	31.91	10.39	78.39
LACR (D=20, Baseline)	**32.63**	13.49	97.00
LACR (D=22)	32.62	14.83	123.00

↑ and ↓ indicate that higher and lower values are better, respectively. # denotes the number of parameters.

## Data Availability

The public sources of the data mentioned in this study are described in the paper.
